# Human ring chromosome registry for cases in the Chinese population: re-emphasizing Cytogenomic and clinical heterogeneity and reviewing diagnostic and treatment strategies

**DOI:** 10.1186/s13039-018-0367-3

**Published:** 2018-02-27

**Authors:** Qiping Hu, Hongyan Chai, Wei Shu, Peining Li

**Affiliations:** 1 Department of Cell Biology and Genetics, School of Pre-Clinical Medicine, Guangxi Medical University, Nanning, Guangxi 530021 China; 2 Laboratory of Clinical Cytogenetics and Genomics, Department of Genetics, Yale School of Medicine, New Haven, CT 06520 USA

**Keywords:** Ring chromosome, Online registry, Ring chromosome instability, Dynamic mosaicism, Genomic imbalances, Syndromic phenotypes, Diagnostic guidelines, Chromosome therapy

## Abstract

**Background:**

Constitutional ring chromosomes are rare orphan chromosomal disorders. Ring chromosome syndrome featuring growth retardation and mild to intermediate intellectual disability is likely caused by the dynamic behavior of ring chromosome through cell cycles. Chromosomal and regional specific phenotypes likely result from segmental losses and gains during the ring formation. Although recent applications of genomic copy number and sequencing analyses revealed various ring chromosome structures from an increasing number of case studies, there was no organized effort for compilating and curating cytogenomic and clinical finding for ring chromosomes.

**Methods:**

A web-based interactive ‘Human Ring Chromosome Registry’ using Microsoft Access based relational database was developed to present genetic and phenotypic findings of ring chromosome cases. Chinese ring chromosome cases reported in the literature was reviewed and compiled as a testing data set to validate this registry.

**Results:**

A total of 113 cases of ring chromosomes were retrieved in all chromosomes except for chromosomes 16, 17 and 19. The most frequently seen ring chromosomes by a decreasing order of relative frequencies were ring 13 (14%), X (12%), 22 (10%), 15 (9%), 14 (7%), and 18 (7%). Genomic imbalances were detected in 18 out of 19 cases analyzed by microarray or sequencing. Variable clinical manifestations of developmental delay, dysmorphic facial features, intellectual disability, microcephaly, and hypotonia were noted in most autosomal rings. Chromosomal specific syndromic phenotypes included Wolf-Hirschhorn syndrome in a ring chromosome 4, cri-du-chat syndrome in a ring chromosome 5, epilepsy in ring chromosomes 14 and 20, Turner syndrome in ring chromosome X, and infertility in ring chromosomes 13, 21, 22 and Y. Effective growth hormone supplemental treatment for growth retardation in a ring chromosome 18 was noted.

**Conclusions:**

Based on findings from these Chinese ring chromosome cases, guidelines for cytogenomic diagnosis and criteria for case registration were proposed. Further research to define underlying mechanisms of ring chromosome formation and dynamic mosaicism, to delineate the genotype-phenotype correlations, and to develop chromosome therapy for ring chromosomes were discussed.

**Electronic supplementary material:**

The online version of this article (10.1186/s13039-018-0367-3) contains supplementary material, which is available to authorized users.

## Background

Constitutional ring chromosomes are a rare type of intra-chromosome structural abnormality with an estimated occurrence of 1 in 50,000 newborns [1]. A ring chromosome is resulted from breakage and fusion at the telomeric or distal regions of both chromosome arms; this circular chromosome replaces one normal chromosome and presents unique mitotic behavior. Structurally, ring chromosomes are divided into two types: a complete ring chromosome without loss of genetic materials by telomere-telomere fusion or an incomplete ring with distal or interstitial deletions and duplications by one or multiple breakage-fusion events [2–4]. Through each cell cycle, ring chromosomes will experience mitotic disturbance induced by ring chromosome instability and result in three different fates of stable ring transmission, ring size changes by one sister chromatid exchange, or interlocked rings by two sister chromatid exchanges. This ring chromosome instability presents a ‘dynamic mosaicism’ with cells showing chromosomal or segmental aneuploidies and thus a portion of cell loss from mitotic arrest and apoptosis [5, 6]. Earlier reports of ring chromosome cases by karyotype analysis observed this dynamic mosaicism but failed to detect segmental or cryptic deletions and duplications due to the limited analytical resolution [7, 8]. The application of fluorescence in situ hybridization (FISH) enabled the differentiation of complete rings from incomplete ones and the monitoring of dynamic mosaicism using various probes targeted to the telomeric, subtelomeric, distal and centromeric regions of the involved chromosome [2, 3, 9]. Recently, genomic technologies such as array-comparative genomic hybridization (aCGH), single nucleotide polymorphism (SNP) chip, and whole-genome sequencing were applied to analyze copy number changes and complex rearrangements and revealed various genomic structures of human ring chromosomes [4, 10, 11].

Patients carrying an autosomal ring chromosome shared certain clinical manifestations including proportional growth retardation, developmental delay, mild to severe intellectual disability, microcephaly, and mild to intermediate dysmorphic facial features. These common manifestations were classified as ‘ring chromosome syndrome’ and were thought to be caused by ring chromosome instability from recognized cases of complete ring chromosome [8, 12]. However, ring chromosome cases with certain phenotypes associated with segmental deletions and duplications had been reported [3, 10]. In clinical practice, ring chromosomes were detected mostly from pediatric patients with growth retardation and occasionally from prenatal cases with intrauterine growth restriction [13, 14]. Ring chromosome X in females with premature ovarian failure and ring chromosome Y in males with azoospermia were noted in adult reproductive clinics [15, 16].

Despite an increase volume of case reports of ring chromosomes from prenatal to postnatal clinics and more detailed cytogenetic and genomic findings with the introduction of genomic technologies, it is still a challenge to interpret the compound effects and to predict the clinical consequences from the dynamic mosaicism and genomic imbalances of ring chromosomes. The rarity of ring chromosome cases demands organized efforts to compile and curate findings from ring chromosome cases for better diagnostic practice and disease classification. This report presented the design and implementation of an online interactive human ring chromosome registry and a comprehensive review of ring chromosome cases in the Chinese population. This initial effort could lead to future collaboration toward the construction of a human ring chromosome atlas as a clinical, research and educational resource for this rare type of chromosomal abnormality.

## Design of an online human ring chromosome registry

**Fig. 1:**
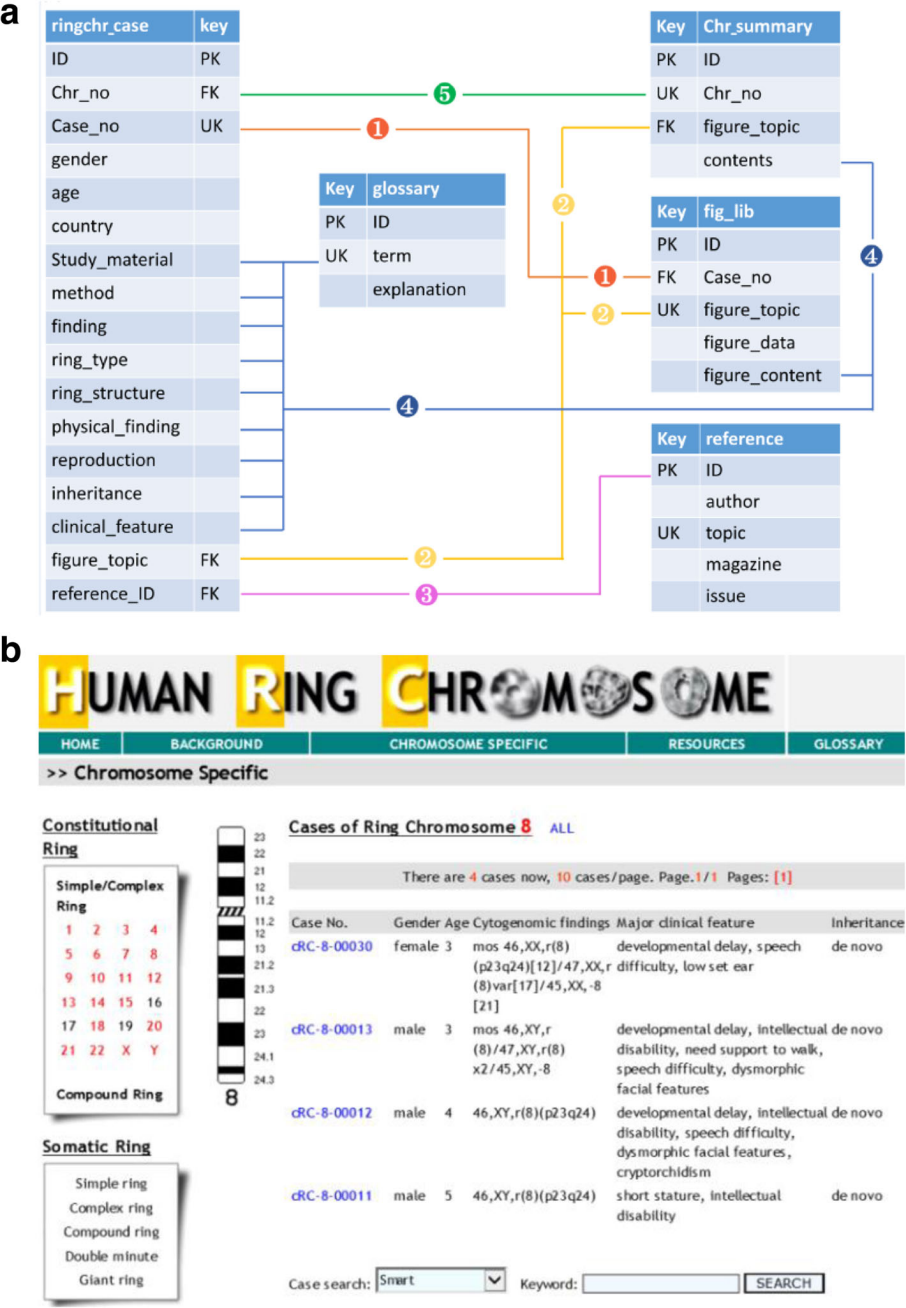
The relational model and website view for the human ring chromosome registry. **a**: The five modules of case, summary, figure, glossary and reference and their relations by numbered and colored lines (PK, UK and FK stand for primary key, unique key and foreign key), **b**: website view of chromosome specific cases in the human ring chromosome registry

A Microsoft Access based relational laboratory information management system had been developed and implemented for a clinical cytogenetics laboratory [17]. Following the design principles for a genetics home reference [18], a Microsoft Access based online interactive registry for human ring chromosomes was developed. This registry includes five modules of case information, figure, summary, reference, and glossary. The case module (ringchr_case) covers an assigned identification number, gender, age, cytogenetic and genomic results, and clinical findings. The figure module (fig_lib) stores illustrative images. The summary module (chr_summary) presents contents and statistics of major clinical features for a specific chromosome. The reference module collects case reports and articles of ring chromosome cases registered in the database. The glossary module contains genetic and clinical terms used in genetic diagnostic and clinical description. Relationships between these modules are determined using primary key (PK), unique key (UK) and foreign key (FK). Figure 1 shows the relationship model of the five modules and a view of the web page for this registry. This ‘Human Ring Chromosome’ registry is loaded online with a website of “http://web.gxmu.edu.cn/shengwu/HRC/home.asp”. To validate the modular relations and functions of this human ring chromosome registry, ring chromosome cases in the Chinese population was collected as the initial testing data set.

## Ring chromosome cases in the chinese population

### Comprehensive review of ring chromosome cases

Case reports and original articles of constitutional ring chromosomes were searched from Chinese medical journals archived in the SinoMed (http://www.sinomed.ac.cn/), VIP (http://qikan.cqvip.com/), CNKI (China National Knowledge Infrastructure, http://www.cnki.net) and WANFANG DATA (http://www.wanfangdata.com) as well as English journals archived in the PubMed (https://www.ncbi.nlm.nih.gov/pubmed/) for a period from 1979 to June 2017. For each case, the gender, age, cytogenetic and genomic results, clinical findings, and family history were retrieved, reviewed and registered into the ring chromosome registry. The relative frequencies of ring chromosomes were calculated by the case number of a specific chromosome divided by the total number of cases. The presence and penetrance of clinical features was shown in a heat map by the observed frequency among cases of a chromosome or all autosomes. Cases with supernumerary small marker chromosomes (sSMC) in the form of a small ring chromosome were excluded because these cases and their cytogenomic and clinical findings were summarized in an online sSMC database (http://ssmc-tl.com/sSMC.html).

### Cytogenetic and genomic results from ring chromosome cases

A systematic search of Chinese ring chromosome cases found a total of 113 cases from 94 case reports and four original articles. The age, gender, study materials and methods, cytogenetic and genomic results, major clinical findings, family history, and references are summarized in the Additional file 1: Table S1. Of these 113 cases, 95 cases were autosomal ring chromosomes except for no cases of chromosomes 16, 17 and 19 while 18 cases were sex chromosome rings. The relative frequencies by a decreasing order were ring chromosome 13 in 14%, ring chromosome X in 12%, ring chromosome 22 in 10%, ring chromosome 15 in 9%, ring chromosomes 14 and 18 each in 7%, ring chromosomes Y, 4, 5, 6, 8, 9 and 21 each in 4%, ring chromosome 2 in 3%, ring chromosomes 3, 7, 10 and 20 each in 2%, and ring chromosomes 1, 11 and 12 each in 1%. The male to female ratio for autosomal ring chromosome cases was 44 vs 51 (Chi-square test, *P* value 0.49), indicating no statistically significant gender bias. However, more female cases in ring chromosomes 13, 18 and 22 were noted. There is a need of more cases to evaluate the sex ratio for individual chromosome. Compound chromosomal abnormalities were noted in one physically normal adult male who was referred by a perinatal infant death and detected with a derivative chromosome 6 from a 6p/13q translocation and a ring chromosome 13 [19], and in an infant with XYY and a ring chromosome 13 [20].

**Fig. 2:**
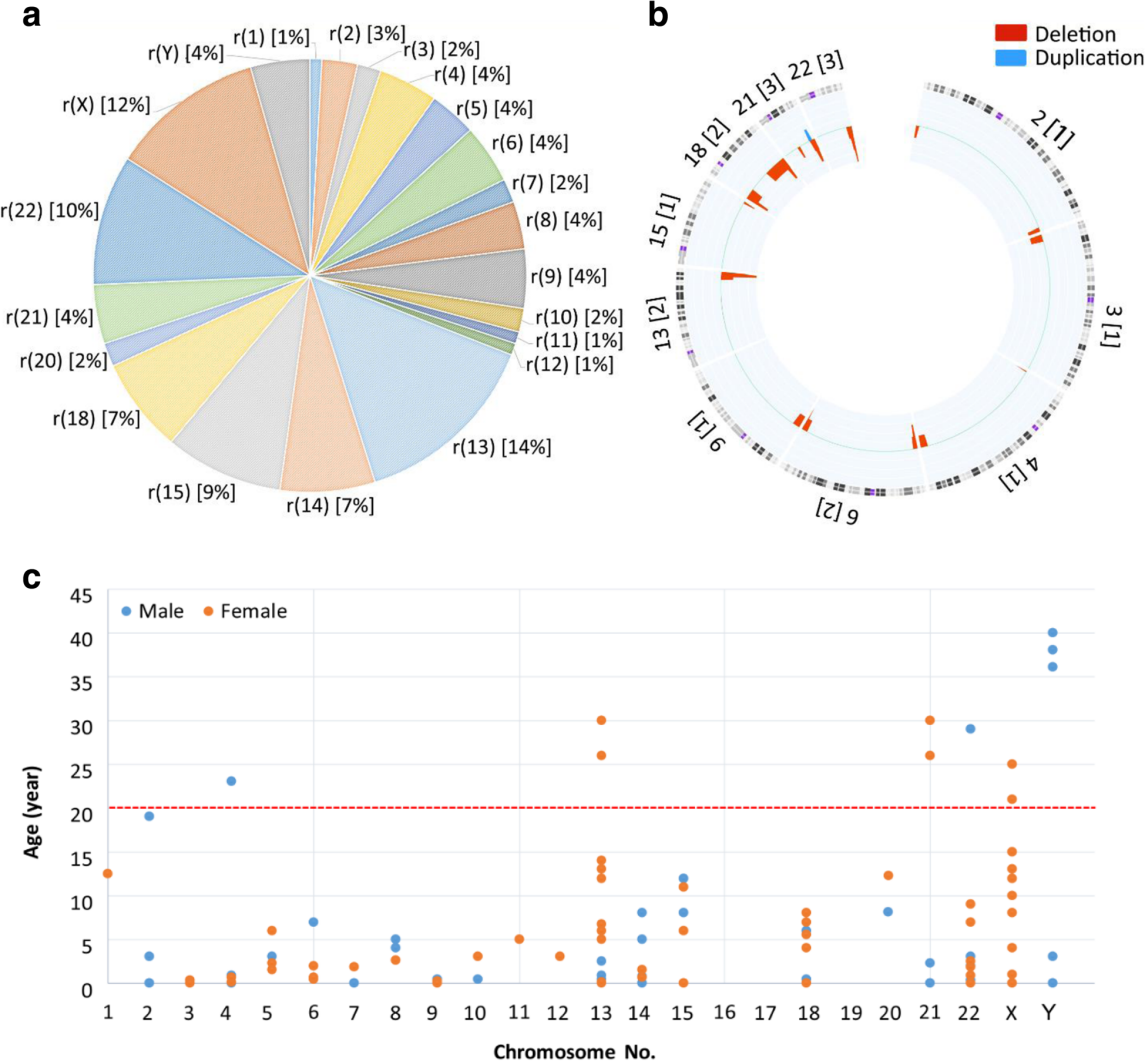
Cytogenomic results of ring chromosome cases in the Chinese population. **a**: A pie chart showing relative frequencies of ring chromosomes in the Chinese cases. **b**: A circus plot showing genomic imbalances detected in ring chromosomes 2, 3, 4, 6, 9, 13, 15, 18, 21 and 22. **c**: The distribution of age, gender and number of cases of ring chromosomes. Each dot represents one case (blue for male and orange for female). Prenatal cases are placed at age zero while pediatric and adult cases are separated by a red dash line at age 20

Regional specific assays (RSA) such as FISH, multiple ligation probe amplification (MLPA), and short-tandem repeats (STR) were applied to 24 cases. Genome-wide aCGH and SNP-chip were used on 17 cases and genomic sequencing was used on two cases. Three out of 13 cases by RSA alone were noted with distal deletions while 18 out of 19 cases by genomic analysis were noted with distal deletions ranging from 160 Kb (kilo base-pair) to 34 Mb (mega base-pair). The latter result suggested that most cases of ring chromosome were incomplete rings although reporting bias of selected cases should be taken into consideration. The relative frequencies, the genomic imbalances, age and gender distribution of reported Chinese ring chromosome cases are shown in Fig. 2.

Dynamic mosaicism was detected in 63 cases by karyotype analysis of 20 to 250 metaphase cells. For cases with autosomal ring chromosomes, ring chromosome variants including ring size changes, interlocked rings, an extra copy of ring, or a derivative chromosome from ring breakage were noted in 2%–17% of cells with an average of 11%; monosomy due to the loss of the ring chromosome was noted in 3%–31% of cells with an average of 13%, and normal karyotype was noted in 1%–84% of cells with an average of 32%. For cases with sex chromosome rings, ring chromosome variants were not seen but losses of ring chromosome X or Y were noted in 9%–93% of cells with an average of 62%. These results indicated that only a small portion of cells can tolerate autosomal ring instability but a relatively larger portion of cells can survive the loss of a sex chromosome ring. However, the lack of diagnostic guidelines specific for analyzing dynamic mosaicism has made it difficult for reliable case-by-case and chromosome-to-chromosome comparisons of ring chromosome instability. In a prenatal case with a ring chromosome 13, cytogenetic analysis showed similar dynamic mosaicism of ring chromosome 13, dicentric ring 13, and monosomy 13 in cultured amniocytes and cord blood leukocytes but a small marker chromosome 13 and monosomy 13 in placenta tissue; aCGH analysis revealed a 4.22 Mb deletion at 13q34 in the ring chromosome 13 and a 91.8 Mb deletion of 13q11-q34 in the marker chromosome. This fetoplacental chromosomal discrepancy suggested a tissue-specific karyotype evolution and the marker chromosome as a residual fragment from ring chromosome rescue [21]. The finding of a marker chromosome in prenatal analysis of chorionic villus specimen should be interpreted cautiously and followed by amniocentesis or cord blood study. Overall, cytogenomic heterogeneity was noted in the high percentage of genomic imbalances in ring chromosome structure, different levels of dynamic mosaicism, and variable karyotype evolution in different tissues.

### Clinical findings in ring chromosome cases

**Fig. 3:**
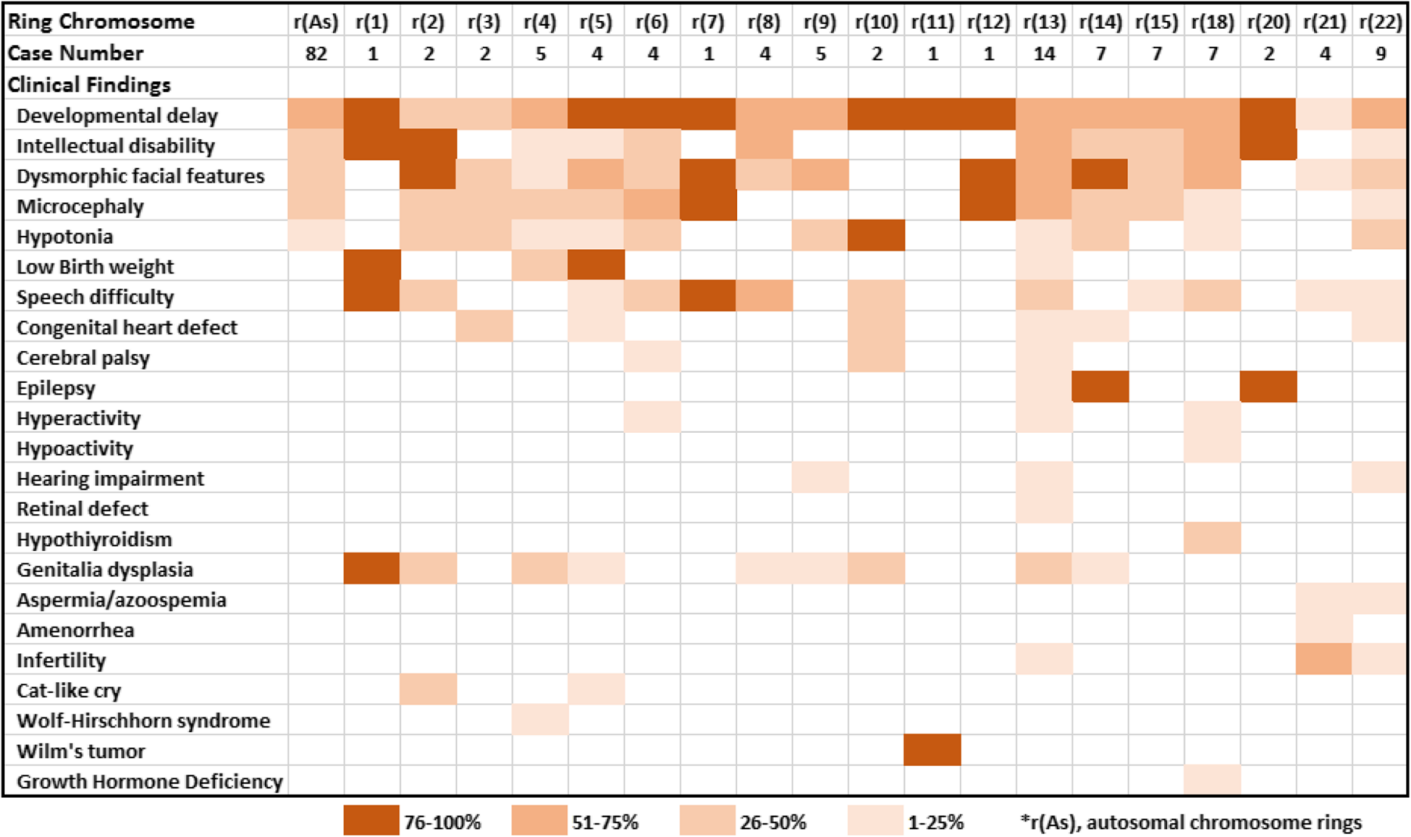
A heat map showing major clinical features in the autosomal ring chromosome cases

Of the 113 cases with a ring chromosome, 89 cases (77 autosomal and 12 sex chromosomal rings) were assessed in perinatal or pediatric clinics, 14 cases (13 autosomal and one sex chromosome rings) were diagnosed prenatally, and 10 cases (six autosomal and four sex chromosome rings) were noted in infertility or reproductive clinics. Major clinical findings compiled from 82 postnatal cases with autosomal ring chromosomes are shown as a heat map in Fig. 3. Despite only one or two cases for several ring autosomes, the most common findings were developmental delay (52/82), dysmorphic facial features (41/82), intellectual disability (31/82), microcephaly (26/82), and hypotonia (18/82). These findings are considered as features of so-called ‘ring chromosome syndrome’ but variable clinical manifestations are obvious. Other frequently seen clinical presentations in different ring autosomes were speech difficulty, genitalia dysplasia in different forms, various types of congenital heart defects, and low birth weight.

Chromosome-specific syndromic phenotypes resulting from segmental imbalances were noted. Features of Wolf-Hirschhorn syndrome (OMIM#194190) known to 4p16.3 deletions was reported in a case with a ring chromosome 4. Cri-du-chat syndrome (OMIM#123450) related with 5p deletions was reported in one case with a ring chromosome 5 and another case with a ring chromosome 2. Wilms tumor caused by heterozygous mutations in the WT1 gene at 11p13 (OMIM#194070) was noted in one case with a ring chromosome 11. Retinal defect was noted in one case with a ring chromosome 13, inferring an increased risk for retinoblastoma (OMIM#180200) caused by deletions and mutations in the RB1 gene at 13q14.2. Epilepsy as a highly penetrant phenotype was noted in all seven cases with a ring chromosome 14 and two cases with a ring chromosome 20. Epilepsy and other symptoms in ring chromosome 14 are referred as ring chromosome 14 syndrome (OMIM#616606). Growth hormone deficiency was reported in one case with a ring chromosome 18; periodic growth hormone supplement treatment was effective [22]. Growth hormone deficiency was a key feature of distal 18q23 deletion with significant treatment implications [23]. For adult patients with infertility, aspermia was noted in two adult males with a ring chromosome 21 or 22, amenorrhea was noted in two adult females with a ring chromosome 21, Tuner syndrome-like phenotype was noted in almost all cases with a ring chromosome X, and azoospermia, aspermia, small testes were seen in three cases with a ring chromosome Y. Overall, clinical heterogeneity for ring chromosomes was indicated by the incomplete penetrance, variable expressivity, and different age of onset of ring chromosome syndrome and the compound effects from chromosome-specific or region-specific phenotypes.

The clinical indications for prenatal diagnosis of ring chromosome cases included ultrasound findings of intrauterine growth restriction, oligohydramnios, microcephaly, lissencephaly, anencephaly, ventricular septal defect, and increased nuchal fold as well as abnormal prenatal screening results for increased risk of Down syndrome. Termination of pregnancy had been an option for fetus with severe anomalies and defined ring chromosome abnormalities. Excluding cases with parental denial or no follow up tests, parental studies performed in 60 families with a proband carrying a ring chromosome documented normal karyotypes for both parents. This result indicated that almost all ring chromosomes are de novo in origin. The clinical presentations from prenatal and postnatal cases with a ring chromosome and their family history could be helpful for genetic counseling.

## Toward precise genetic diagnosis and clinical management

With annual births of 17.86 million in China for 2016 and the occurrence of 1/50,000 for a constitutional ring chromosome, there are an approximately 350 newborns with this group of rare orphan chromosomal disorders each year in the Chinese population. A comprehensive review of medical literature found only 113 reported Chinese cases with a ring chromosome which probably represented about 1% of total cases for the past four decades in the Chinese population. Even though only a small percentage of ring chromosome cases were published, this case series was by far the largest in an ethnic group. The findings from these cases provided insights into developing practice guidelines and research collaboration for genetic diagnosis, clinical management, and disease treatment.

### Considerations of diagnostic guidelines for constitutional ring chromosomes

Integrated analyses by karyotype, FISH and SNP chip on a series of 14 cases of autosomal ring chromosomes detected the dynamic mosaicism of ring chromosome variants in 5%–16.3% of the 300 metaphase cells examined and distal deletions in the range of 364 Kb to 18 Mb in 12 cases [24]. The range of dynamic mosaicism and the portion of genomic imbalances in these 14 ring chromosome cases were consistent with findings from this Chinese case series. The analysis by aCGH and SNP chip can delineate the genomic imbalances in the ring chromosome, map critical regions containing candidate genes for certain phenotypes, and infer underlying gene dosage or position effects [10, 25]. A study of 27 patients with a ring chromosome 14 mapped the retinal abnormality and epilepsy within the proximal 14q11.2-q12 region containing the retinitis pigmentosa gene *RPGRIP1*, the neural retina leucine zipper gene *NRL*, and the *FOXG1* gene. It was speculated that the formation of the ring could induce the spreading of heterochromatinization and dysregulate the gene expression [26]. Dysregulation of *FOXG1* gene by an acentric ring chromosome 14 was noted in a patient with epilepsy [27]. Based on the clinical types per cytogenomic findings and reproductive patterns from male and female carriers per family history, recommendations for cytogenomic analysis specific for ring chromosome 21 was proposed [4]. Currently, except for the general cytogenetics guidelines and standards developed by American College of Medical Genetics and Genomics or other regional professional organizations, there were no diagnostic consensus and guidelines designated for analyzing the genomic structures and measuring the dynamic mosaicism from ring chromosomes. For all constitutional ring chromosomes, an integrated cytogenomic approach should be implemented for three purposes: 1) differentiating complete rings from incomplete ones, 2) delineating genomic imbalances in the ring chromosomes, and 3) defining levels of ring chromosome instability. The diagnostic guidelines for this approach should include the following:

- Routine chromosome analysis on a peripheral blood specimen should be performed on 100 metaphases for percentages of cells with typical ring chromosome, ring variants, and ring loss. FISH tests should be performed on 300 directly prepared interphase cells using chromosome-specific subtelomeric, centromeric, and targeted locus-specific probes to access distal deletions/duplications, ring variants and ring loss. This analysis of 100 to 300 cells will allow an accurate detection of dynamic mosaicism in 2%–3% of cells with a 95% confidence level [28]. If possible, the analysis of different tissues to further define the mosaic pattern should be considered.
- Genomic analysis by aCGH, SNP chip, or genomic sequencing should be performed to characterize the distal or interstitial copy number imbalances in the ring chromosomes. The result from this DNA-based genomic analysis should be correlated with findings from cell-based chromosome and FISH tests.
- Evidence-based interpretation should be provided regarding clinical significance for detected dynamic mosaicism and genomic imbalances of ring chromosomes. The implications on physical and mental development as well as risks for cancer and reproduction should be addressed [29]. Evidences for genomic imbalances could be collected from similar cases reported in the literature and databases of linear copy number variants. These databases include Database of Genomic Variants (DGV), ClinVar, and DatabasE of Chromosomal Imbalance and Phenotype in Humans using Ensembl Resources (DECIPHER). Tracks of these databases could be linked from the Human Genome Browser (http://genome.ucsc.edu/).
- Follow-up parental study should be considered to determine de novo or familial transmission of the ring chromosome. It was estimated that inherited ring chromosomes exist in approximately 1% of ring chromosome cases; familial cases usually presented relatively mild clinical manifestations but one third of the transmitted offspring were more severely affected [30].

**Fig. 4:**
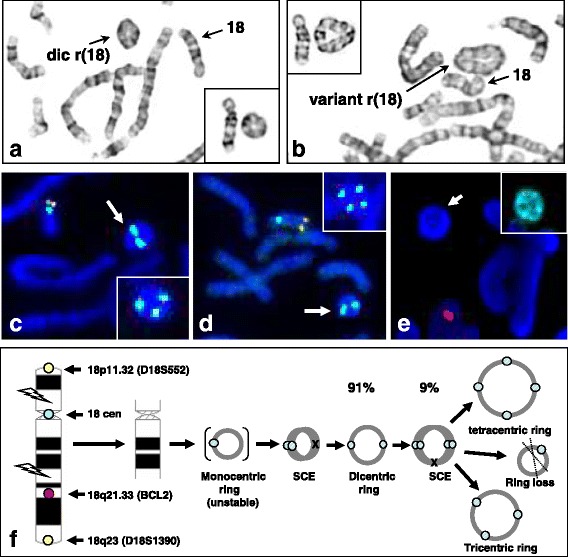
Patterns of dynamic mosaicism and karyotype evolution in a dicentric ring chromosome 18. **a**/**b**: Karyotype showing a dicentric ring chromosome 18 and variant ring chromosomes 18. **c**/**d**: FISH images showing absence of subtelomeric signals in the dicentric ring chromosome 18 and variant tricentri and tetracentric rings (in inset). **e**: FISH image showing absence of BCL2 locus in the ring chromosome (paint ring chromosome 18 in inset). **f**: a diagram showing dynamic mosaicism induced by sister chromatid exchange (SCE). Likely unstable monocentric ring 18 was not seen by karyotyping and FISH. Dicentric ring chromosome 18 was seen in 91% of cells and variants of tri−/tetra-centric ring chromosomes 18 and ring loss was seen in 9% of cells

There is an urgent need for consensus on defining level of ring chromosome instability by observed dynamic mosaicism. This report and a recent case series documented a range of 2%–17% for autosomal ring chromosome variants and ring loss [24]. A study of ring chromosome 20 cases found that the mosaic ratio of r(20) was directly correlated with the severity of cognitive impairment and inversely correlated with the onset age of epilepsy; this finding should be interpreted with caution since the level of mosaicism in blood may not reflect the level of mosaicism in other tissue, especially the brain [31]. A study of aneuploidy in the developing human brain determined an average aneuploidy frequency as 1.25–1.45% per chromosome and thus an estimated overall aneuploidy percentage of 30–35% for all chromosomes; these findings revealed confined chromosomal mosaicism in the brain and suggested a potential link between chromosome instability and human brain diseases [32]. These studies provided evidences on the importance of defining levels of ring chromosome instability. The low, intermediate and high levels of ring chromosome instability may be defined using an arbitrary measurement of less than 5%, 6–10% and more than 10% of metaphase/interphase dynamic mosaicism, respectively. For example, the analysis of 100 metaphase cells from a case (unpublished) revealed a primary pattern of a dicentric ring chromosome 18 in 91% of cells and secondary variant patterns of tri−/tetra-centric rings and monosomy 18 likely due to the loss of ring in 9% of cells. This could be considered as an intermediate level of ring chromosome instability. The cytogenetic results and the karyotypic evolution for this dicentric ring chromosome 18 are shown in Fig. 4. Recent progresses in the use of genomic analysis as the first-tier genetic testing and non-invasive prenatal screening of aneuploidies by sequencing of maternal serum cell-free DNA have improved the diagnostic efficacy for a wider spectrum of cytogenomic abnormalities [33–35]. However, for analyzing structural rearrangement like ring chromosomes, integrating cell-based karyotype and FISH analyses with DNA-based genomic analysis should be the gold standard.

### General recommendations for clinical management of ring chromosome cases

Cases of ring chromosome presents a spectrum of clinical features caused by ring chromosome instability and additional features compounded by dosage effect from genomic imbalances, position effect from structural rearrangement, or changes in epigenetic modification [5, 36]. Of the 113 cases in this report, approximately 12%, 79% and 9% of them were detected from prenatal, pediatric and reproductive clinics, respectively. Clinical features of ring chromosome syndrome and chromosome-specific syndromic phenotypes were presented. Variable clinical manifestations in ring chromosome cases were noted in one group with severe clinical symptoms and the other group with no obvious clinical problems apart from infertility [24]. Effective supplemental treatment of growth hormone deficiency in a ring chromosome 18 was noted [22]. The general principles for clinical management of ring chromosome cases should include: 1) thorough clinical assessment of developmental delay, intellectually disabilities, speech difficulties, facial features, ocular anomalies, epilepsy, genitalia dysplasia, and other related symptoms, 2) identifying medically actionable symptoms and providing targeted therapy and follow up treatments, 3) delivering genetic counseling to parents of the affected with detailed information about the disease progression and related risks in cancer predisposition and reproduction failure.

An ad hoc task force proposed guideline recommendations for clinical diagnosis and management of ring chromosome 14 syndrome [37]. These guidelines were based on data from peer-review scientific literature and on a subsequent holistic summary by a heterogeneous panel of experts. For the major symptoms of epilepsy, hypotonia, recurrent infections, vision and hearing complications, respiratory complications, and communication and language disorders seen in patients with ring chromosome 14, recommendations for general management and specific treatments of each symptom were generated. For example, epilepsy should be treated from the onset with anticonvulsive therapy. Advices for taking care of a child with this rare and complex syndrome were offered to parents. This chromosome-specific symptom-oriented disease management and treatment set a model for cases involving other ring chromosomes.

For registering cases into the implemented human ring chromosome registry, a task force by clinical cytogeneticists and geneticists will be organized to review cytogenomic findings following the diagnostic guidelines and to curate clinical findings. Accumulation of more ring chromosome cases with defined genomic structures and dynamic mosaicism and detailed clinical manifestations will provide better genotype-phenotype correlation for predicting clinical consequences and planning treatments.

## Understanding the biology of human ring chromosomes

**Fig. 5:**
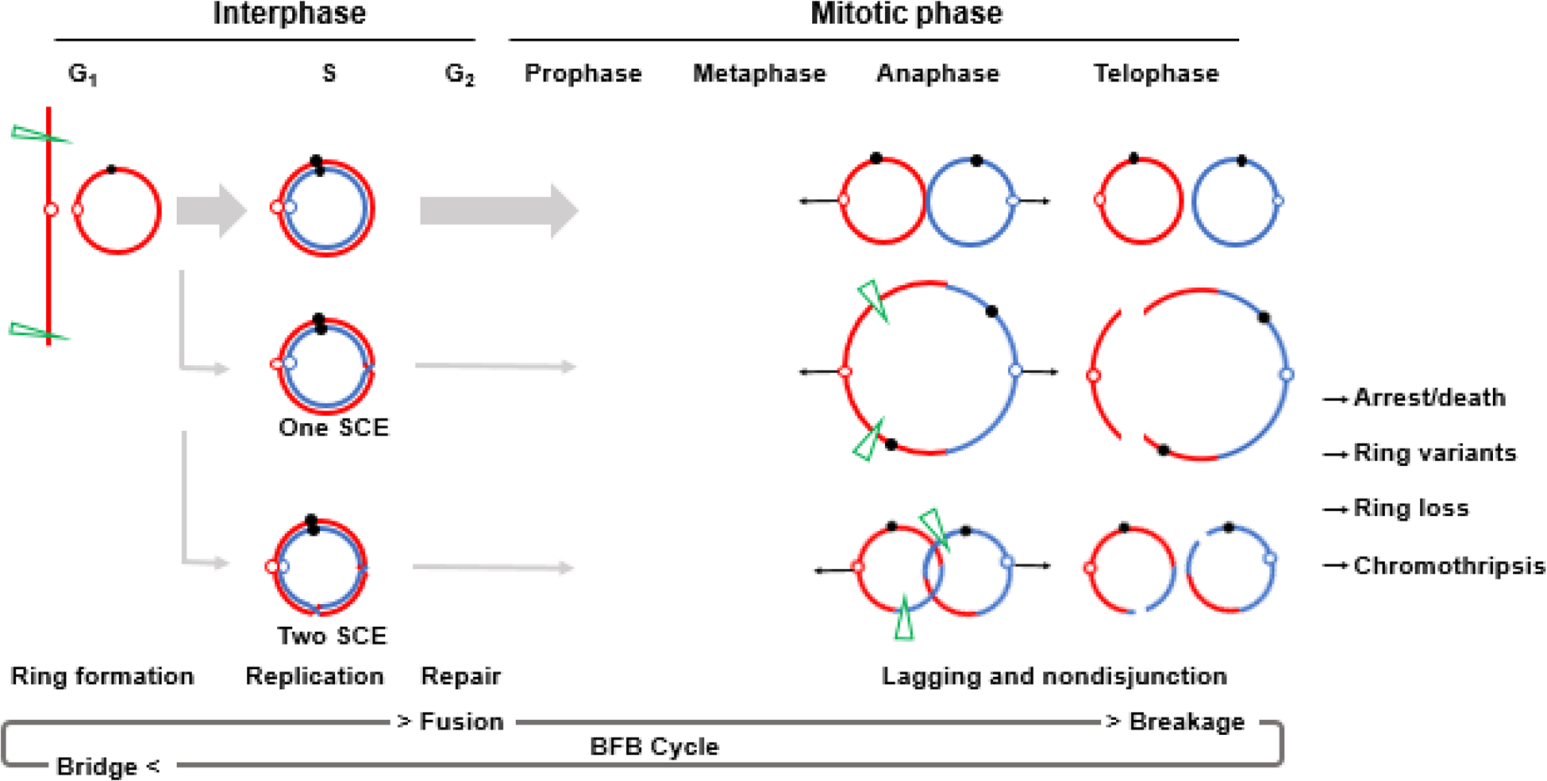
Ring chromosome formation and dynamic mosaicism by breakage-fusion-bridge cycle through a cell cycle

It has been thought that human ring chromosomes are formed by a starting breakage-fusion event at the distal or telomeric ends of both arms [24] and then go through breakage-fusion-bridge (BFB) cycles for additional segmental imbalances in the ring or even chromothripsis to form a ring with randomly reassembly of multiple broken segments [38, 39]. The breakage-fusion point can be detected by aCGH or SNP chip analysis but the jointed sequences and underlying fusion mechanisms remain largely unknown. This ring chromosome replicates in the S phase with none, one, or two sister chromatid exchanges to generate intact ring, dicentric ring, or interlocked rings, respectively. During the mitotic phase, the dicentric or interlocked rings require breakage and thus show lagging at anaphase and nondisjunction into telophase. A dicentric chromosome can persist through mitosis and cytokinesis by forming a long chromatin bridge coated with nuclear membrane between daughter cells; this bridge resolves into single strand DNA by the cytoplasmic 3′ nuclease TREX1 and induces nuclear envelope rupture [40]. Mis-segregated or nondisjunction chromosomes with various types of genomic rearrangements including chromothripsis are captured in a micronucleus [38]. DNA within micronuclei could go through replication in S-phase and repairing in G2 phase. High rate of cell deaths caused by mitotically unstable ring chromosome variants has been suggested for the evolution of cell lines with different ring chromosome components [41]. Somatic ring chromosomes have been seen in various types of cancers; chromothripsis in which multiple copy number gains or losses confined to one or a few chromosomes could contribute to the formation of somatic ring chromosomes [39]. Figure 5 shows the breakage-fusion-bridge cycle for a ring chromosome through a cell cycle. It was hypothesized that the anaphase lagging and resultant aneuploids could induce cell cycle arrest and cell death. Furthermore, it is hypothesized that there may be a selection process for karyotype evolution through a few early cell cycles to realize a ring structure sufficient for cell survive and tolerable for ring instability. To test these two hypotheses, methods for cell-based tracking and monitoring of ring chromosome behavior and cell fates and DNA-based targeted sequencing of various ring chromosomes should be developed.

It was suggested that larger ring chromosomes showed significantly more instability than small rings [8]. This correlation between ring instability and the size of the chromosome was partly reflected from the relative frequencies of ring chromosomes. The most frequently seen ring autosomes in the Chinese cases were chromosomes 13, 22, 15, 14, 18 and 21. Dynamic mosaicism from these ring chromosomes diverted a portion of cells with aneuploidy of the involved chromosome. As a comparison, the most frequently seen aneuploidy in products of conception were trisomies 13, 15, 16, 18, 21 and 22 [35]. There is an overlap in most of these small size autosomes. However, the absence of ring chromosomes 16, 17 and 19 in the reported Chinese cases suggested that, in addition to size, the content of a chromosome could affect the survive of cells or even the fetus. Cell lines from cases of ring chromosomes could be used as an in vitro cellular model for genetic mosaic analysis of chromosomal or segmental aneuploidy to understand the underlying mechanisms of chromosomal number control and the impact on human development and disease.

Reprograming human fibroblasts containing ring chromosomes 13 and 17 to induced pluripotent stem cells (iPSC) discovered the correction of ring chromosome through a compensatory uniparental disomy (UPD) mechanism [42]. This cell-autonomous correction involved first the loss of ring chromosome and then the duplication of the normal chromosome in five to ten cell culture passages; the correction ratio varied from different iPSC clones. A potential strategy for chromosome therapy to correct chromosome abnormalities using ring chromosome was proposed [43]. Theoretically, loxP cassettes could be inserted into the p and q arm of an abnormal chromosome via CRISP-Cas9. Treatment with Cre-mediated recombinase will induce ring chromosome formation at the loxP loci. Cells containing the newly formed ring chromosome are grown for several passages to induce ring loss and then trigger compensatory UPD for monosomy rescue. This strategy could also be used to reduce trisomy to disomy by induced ring loss and to correct pathogenic CNVs or large aberration by compensatory UPD. Limitations in this chromosome therapy concept include validity and efficacy of the technical procedures, the risk of exposing recessive disease or imprinting disorders, and ethical considerations. Chromosome mosaicism is a relatively common finding in in vitro fertilization derived human embryos. Trisomy rescue and monosomy compensatory and resultant UPD have been documented in the prenatal findings of fetoplacental discrepancy and confined placenta mosaicism. Self-correction of chromosomal abnormalities in human preimplantation embryos and embryonic stem cells has been explained by increased death and decreased division of aneuploid cells or allocation of the aneuploidy in the trophectoderm [44, 45]. In a small case series, intrauterine transfer of mosaic aneuploid blastocysts developed into healthy euploid newborns [46]. However, if compensatory UPD is truly a cell-autonomous process, cases with ring chromosomes will showed self-corrected cells with normal disomic pattern for the involved chromosome. Of the 95 cases with an autosome ring in this report, only nine cases were noted with normal cells and there was no study to determine a true mosaicism or a compensatory UPD. A study of 16 cases with ring chromosome 14 noted biparental inheritance and excluded UPD [26]. Therefore, the cytogenetic results did not observe a large-scale in vivo self-correction. Cellular reprogramming for iPSC may be a necessary step to trigger compensatory UPD. Further study to understand the mechanisms of ring chromosome loss and compensatory UPD is needed for practical chromosome therapy.

## Future directions

The implemented human ring chromosome registry can play important roles in facilitating genetic diagnosis and developing translational research. With the formation of a task force by clinical cytogeneticists and geneticists, criteria and procedures for registering, compiling and curating cases into this human ring chromosome registry will be formulated; evidence-based diagnostic guidelines and management recommendations for ring chromosome patients will be proposed. Easy access to compiled and curated diagnostic and clinical data as well as properly preserved residual specimens are the key prerequisites for promoting collaborative research. Clinical cytogenetic laboratories should consider registering cases into the human ring chromosome registry and saving residual specimens. Standard operating procedures for proper biobanking of residual specimens should be developed and ethical and legal considerations for research applications should be resolved [47].

## Conclusions

In summary, an online human ring chromosome registry was designed and implemented to review cytogenomic results and clinical manifestations from a set of ring chromosome cases reported in the Chinese population. Relative frequencies, age and gender distributions, and various phenotypes of all ring chromosomes except for chromosomes 16, 17 and 19 were revealed. Cytogenomic heterogeneity was noted in variable genomic imbalances in ring chromosomes, different levels of dynamic mosaicism, and possibly tissue-related karyotype evolution. Human ring chromosomes showed heterogeneous clinical findings ranging from intrauterine growth restriction, postnatal developmental delay and multiple congenital anomalies, adult reproduction failure, and risk for cancer. A framework of organized efforts for translational and basic research collaboration to define underlying mechanisms of ring chromosome formation and dynamic mosaicism, to delineate the genotype-phenotype correlations, and to develop chromosome therapy for ring chromosomes is under consideration.

## Additional file

Additional file 1: **Table S1.** Chinese ring chromosome cases reported in the literature (XLSX 69 kb)

## Abbreviations

aCGH: Array comparative genomic hybridization
BFB: Breakage-fusion-bridge
CNKI: China National Knowledge Infrastructure
CNV: Copy Number Variants
DECIPHER: Database of Chromosomal Imbalance and Phenotype in Humans using Ensemble Resources
DGV: Database of Genomic Variants
FISH: Fluorescence in situ hybridization
FK: Foreign key
iPSC: Induced pluripotent stem cells
Kb: Kilo base-pair
Mb: Mega base-pair
MLPA: Multiple ligation probe amplification
OMIM: Online Mendelian Inheritance in Man
PK: Primary key
RSA: Regional specific assays
SNP: Single nucleotide polymorphism
sSMC: Supernumerary small marker chromosomes
STR: Short-tandem repeats
UK: Unique key
UPD: Uniparental disomy
